# Effects of Meditation on Cardiovascular and Muscular Responses in Patients during Cardiac Rehabilitation: A Randomized Pilot Study

**DOI:** 10.3390/jcm11206143

**Published:** 2022-10-18

**Authors:** Maximilian E. Rudlof, Boštjan Šimunić, Bianca Steuber, Till O. Bartel, Ruslan Neshev, Petra Mächler, Andreas Dorr, Rainer Picha, Karin Schmid-Zalaudek, Nandu Goswami

**Affiliations:** 1 Department of Physiology, Otto Loewi Research Center for Vascular Biology, Immunology and Inflammation, 8010 Graz, Austria; 2 Institute for Kinesiology Research, Science and Research Centre Koper, 6000 Koper, Slovenia; 3 Rehabilitation Center for Cardiovascular Disease, 8061 St. Radegund, Austria

**Keywords:** cardiovascular diseases, psychosocial stress, transcendental meditation, cardiac rehabilitation, tensiomyography

## Abstract

Background: Cardiovascular diseases are the world’s number one cause of death, with exceeding psychosocial stress load being considered a major risk factor. A stress management technique that has repeatedly shown positive effects on the cardiovascular system is the Transcendental Meditation (TM) technique. The present pilot study aimed to investigate the potential effect of TM on the recovery of cardiac patients. Objectives: We hypothesized that practicing TM in patients undergoing a 4-week cardiac rehabilitation program augments the recovery of cardiovascular parameters and reduces skeletal muscle tone after rehabilitation. Methods: Twenty cardiac patients were recruited and randomly assigned to either the control or the TM group. Cardiovascular parameters were assessed with the Task Force Monitor (TFM) and skeletal muscle contractile properties by Tensiomyography during a sit-stand test, performed at the beginning and end of a 4-week in-patient rehabilitation program. Results: Systolic blood pressure (SBP) was significantly lower after 4 weeks of cardiac rehabilitation, while the RR-interval (RRI) significantly increased. At the skeletal muscle level, the contraction time and maximal displacement increased, though only in the gastrocnemius medialis and biceps femoris muscles and not in vastus lateralis. Group interactions were not observed for hemodynamic parameters nor for muscle contractile properties. Discussion: Although significant improvements in hemodynamic and muscular parameters were observed after 4 weeks of rehabilitation, we could not provide evidence that TM improved rehabilitation after 4 weeks. TM may unfold its effects on the cardiovascular system in the longer term. Hence, future studies should comprise a long-term follow-up.

## 1. Introduction

Cardiovascular diseases (CVD) are the leading cause of death globally [1]. Interestingly, investigation has shown that the incidence of coronary events can be lowered by more than 80% just through lifestyle modifications [2]. In the Interheart study, which included more than 30,000 people from across the globe and was published in the Lancet in 2004, nine parameters were identified that represent about 90% of all modifiable risk factors associated with myocardial infarction [3]. Beyond smoking, dyslipidemia, hypertension, diabetes, obesity, unhealthy nutrition, alcohol consumption, and physical inactivity, psychosocial stress was defined as one of these nine modifiable factors. Further research has revealed that approximately three-fourths of patients suffering from coronary diseases are burdened with high levels of psychosocial stress load [4].

Strategies and habits for stress reduction, such as the practice of meditation have become increasingly popular. Certain meditation techniques have been studied since the 1970s and revealed positive effects on both physical and mental health. Hence, some types of meditation are now classified as integrative therapy approaches that can be applied additionally to guideline-based therapy to augment therapeutic success [5,6].

One of the best-studied techniques is Transcendental Meditation (TM). It derives from an ancient Vedic tradition and was brought to the West in the 1950s by the so-called Maharishi Mahesh Yogi [7]. It is systematically taught exclusively by trained professionals in a standardized procedure. Since then, more than five million people have been instructed about this certain technique (2001) [8]. Up until 2012, more than 600 studies about TM were conducted [8]. Investigations conducted with regard to the potential effects of TM on the cardiovascular system found that it can reduce hypertension [9,10,11,12,13,14], increase heart performance and delay the onset of myocardial ischemia [15,16,17,18], reduce vascular arteriosclerosis [19], lower plasma levels of stress hormones [15,20,21,22,23], support the cession of substance abuse [24,25], and finally reduce overall morbidity and mortality [26,27,28,29].

While most of the research conducted so far has only illuminated the effectiveness of TM in disease prevention and therapy, little literature exists investigating whether TM could positively affect cardiovascular rehabilitation as well.

Considering skeletal muscle tone, meditation has been shown to reduce skeletal muscle sympathetic nervous activity [30]. Although, it is known that muscle stiffness/tone and composition (percent of slow muscle fibers) are related to blood pressure levels [31] and hypertension [32], no study has yet studied the effects of TM on skeletal muscle contractile properties.

## 2. Objectives

The aim of the present study was to evaluate the effectiveness of TM, practiced during and in addition to a 4-week in-patient cardiac rehabilitation program, on hemodynamic parameters and skeletal muscular contractile properties of cardiac rehabilitation patients. We hypothesized that practicing TM for a duration of 4 weeks, in addition to the standard cardiac rehabilitation program, augments the recovery of hemodynamic parameters (heart rate, blood pressure, etc.) and reduces skeletal muscle tone. Our results should form a foundation for further investigation and enable reasonable study settings.

## 3. Materials and Methods

The study was carried out in October and November 2019 at the Cardiac Rehabilitation Clinic in St. Radegund, a facility of the Austrian retirement insurance PVA (Pensionsversicherungsanstalt). The clinic provides rehabilitation programs for patients suffering from cardiovascular events. This prospective pilot study was designed as a randomized controlled trial, with a control and an intervention group. The participants were randomly assigned to one of the two groups to achieve possibly homogenous cohorts (Table 1). Randomization was performed using an online tool for block randomization, providing equal-sized groups for registered patients meeting all inclusion criteria. The measurements (baseline and post-rehabilitation) were performed at fixed times (9:00, 10:30, 13:00, 14:30, and 16:00) and individually repeated at the same time (pre- and post-intervention/control).

Over a period of 2 weeks, all patients arriving at the clinic to start their 4-week rehabilitation program and who met the inclusion criteria (age between 40 to 80 years, prior cardiac event such as myocardial infarction (MI), acute coronary syndrome (ACS), percutaneous coronary intervention (PCI), coronary artery bypass graft (CABG)) were asked to participate in the study. All participants gave verbal and written informed consent and all research was performed in accordance with the Declaration of Helsinki (1989) of the WMA (the World Medical Association). The study was approved by the ethics committee of the Medical University of Graz (EK 31-443 ex 18/19) and preregistered as a pilot study (Nr. NCT05035758; TMY_Rehab) at clinicaltrials.gov. In total, 20 patients were included and randomly assigned to the study TM group or control group, with ten patients each at the beginning.

Control group: participants assigned to the control group solely received the standard rehabilitation therapy program. After the initial health check, all patients received their schedule for the standard rehabilitation program including physical therapy sessions (such as physiotherapy, massages, lymph drainages, electrotherapy, etc.), psychological assistance, and educational seminars. Further, they attended various physical exercise classes and further medical tests were conducted if necessary (Table 1).

TM group: in addition to the standard rehabilitation therapy, two meditation sessions of 20 min per day were added to the schedule. The first weekend was used to introduce participants to the technique of transcendental meditation (TM). Therefore, two TM professionals of the “Austrian society of Maharishi Vedic sciences” (Österreichische Gesellschaft für Maharishi Vedische Wissenschaft) held an introduction seminar in which the participants learned the technique. Afterward, participants were able to practice the meditation on their own. The morning meditation was performed at 7 am before breakfast and the second session at 5 pm in the afternoon. The meditation was performed during the participants’ spare time to avoid overlapping with the rehabilitation schedule. To provide a quiet place for meditation, each morning and evening the vestry and the library of the clinic were reserved for study participants only. Furthermore, a supervision meeting with the meditation teachers took place every week to resolve questions or issues concerning the meditation.

One day after admission to the rehabilitation clinic the first measurement (baseline measurement) was conducted. Four weeks later, at the end of the rehabilitation program, the second (post-rehabilitation) measurement was performed. The measurements were at fixed times (9:00, 10:30, 13:00, 14:30, and 16:00) and individually repeated at the same time of the day to avoid inter-individual physiological variabilities. After assessing body mass and height, a tensiomyographic evaluation of the three muscles postural proximal vastus lateralis (VL), non-postural biceps femoris (BF), and postural-distal gastrocnemius medialis (GM) of the dominant leg was performed in a supine or prone position. Detailed description of the tensiomyographic assessment is available elsewhere [33]. Briefly, in each muscle two maximal tensiomyographic responses to single square 1 millisecond electrical impulses were assessed. From each response, an amplitude (Dm) and contraction time (Tc) from 10% to 90% Dm were calculated, and an average value was taken for further analysis. The cardiovascular parameters were recorded during a supine-to-stand test (STS) with the Task Force Monitor^®^ (TFM) (CNSystems, Graz, Austria), which non-invasively measures cardiovascular hemodynamics and autonomic nervous system activity. The hemodynamic monitoring includes the measurement of blood pressure (upper arm oscillometry and finger plethysmography), heart rate (3-lead ECG), and thoracic impedance (impedance electrodes). The assessment of these basic parameters allows for the calculation of further cardiac parameters such as the RRI (RR-interval), MAP (mean arterial pressure), SV (stroke volume), SI (stroke volume index), CO (cardiac output), CI (cardiac index), TPR (total peripheral resistance), and TPRI (total peripheral resistance index). The assessment itself took approximately 15 min in total. During the first five minutes, participants remained relaxed in a supine position (baseline assessment). Thereafter, participants were instructed to stand up as quickly as possible and remain standing (standing assessment) for a duration of five minutes, until finally they were asked to lay down again for a further five minutes (recovery assessment). During the whole measurement, participants were required to stand or lie still, trying not to move or speak and relax. Further, a calm and quiet environment was essential for this test to be able to collect valid data.

**Figure 1:**
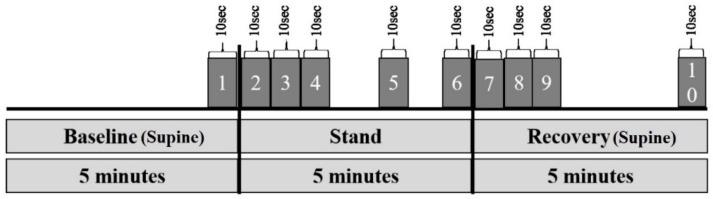
Schematic illustration of the “epochs” used for data generation by the TFM (Brix et al. [34]).

For the statistical analyses, the five minutes of the baseline, standing, and recovery phases were spitted into epochs of 10 s, which were representatively taken into account (Figure 1). The analysis of the data and graphic generation were performed with the programs SPSS (version 26), MATLAB (version 2016A), and Microsoft Excel. All cardiovascular parameters, but not tensiomyography, were log-transformed prior to analysis to ensure a normal distribution, which was checked for by Shapiro–Wilk and Kolmogorov–Smirnov normality tests. Further, all parameters passed Levene’s test of homoscedasticity. Hemodynamic parameters were analyzed by repeated measures ANOVA including pre/post (see Figure 1) as repeated-measures factors and group (control, TM) as between-subjects factor. Similarly, tensiomyographic data were analyzed by a three-factorial general linear model with time (pre, post), muscle (VL, GM, and BF), and group (control, TM) as the three between-subjects factors. Significant effects of, or interactions with time were further analyzed by post hoc tests for each muscle separately to identify differences with time. A Pearson correlation coefficient was calculated for baseline correlations between tensiomyographic parameters and SBP and any subsequent changes in tensiomyographic parameters or SBP.

## 4. Results

### 4.1. Cardiovascular Parameters

Baseline (pre) and post-rehabilitation (post) measurements: In both groups (control and TM group) nine out of ten participants completed the baseline (pre) and the post-rehabilitation measurement (post). Outliers or epochs with poor data quality were excluded from the single analyses. The patients’ characteristics are summarized in Table 1. No significant inter-group differences were found with regard to age, height, or BMI, though participants of the control group had on average a higher weight (*p* = 0.044). The possible covariates of age, weight, and height did not show any significant effects on cardiovascular parameters and were therefore excluded from further analyses. Furthermore, the participant’s sex was also not considered in the analysis since there was only one woman in each group.

**Figure 2:**
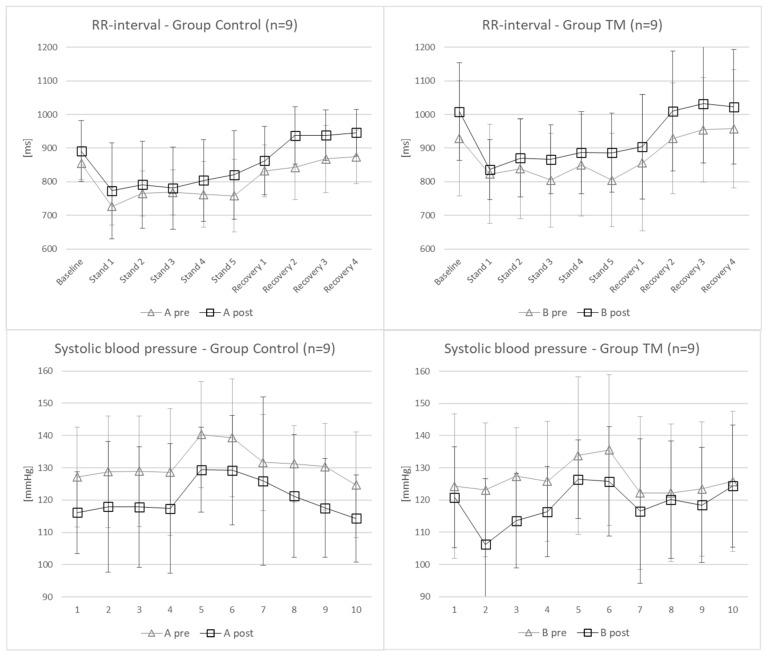
The cardiac parameters RR interval and SBP with significant changes between pre- and post-measurement.

The results of the hemodynamic parameters are summarized in Table 2. Of note, analyses are based on log-transformed data, while raw data are reported. Significant changes from pre- to post- measurement were found for the SBP (*p* = 0.018) and RRI (*p* = 0.05) (Figure 2), while HR nearly missed significance (*p* = 0.058). Thus, SBP and HR decreased while RRI increased after 4 weeks of rehabilitation. However, no interactions between the groups were found. Significant differences between individual epochs are not reported here as these are obvious and well-known phenomena when standing up from a lying/supine position.

### 4.2. Tensiomyographic Parameters

There were no baseline differences in both tensiomyographic parameters. For Tc we found time (*p* < 0.001; η^2^ = 0.866) and time × muscle (*p* = 0.021; η^2^ = 0.320) effects, but not time × group (*p* = 0.493) nor time × group × muscle (*p* = 0.355) effects. Post hoc analysis revealed 19.2% increased Tc in GM (*p* < 0.001) and 21.8% in BF (*p* < 0.001) muscles at post when compared to pre, independent of groups (Table 2). For Dm we found time (*p* < 0.001; η^2^ = 0.756) and time × muscle (*p* < 0.001; η^2^ = 0.557) but not time × group (*p* = 0.115) nor time × group × muscle (*p* = 0.302) effects. Post hoc analysis revealed 47.4% increased Dm in GM (*p* = 0.008) and 45.1% in BF (*p* < 0.001) muscles at post when compared to pre, independent of groups (Table 3).

After pooling the baseline data of both groups, we found negative Pearson correlations in GM (Tc and Dm) and BF (Dm) with SBP measured during standing (Table 4). No correlation was found between tensiomyographic parameters and dBP or mBP, although for mBP correlations were close to significant (0.05 < *p* < 0.10).

**Figure 3:**
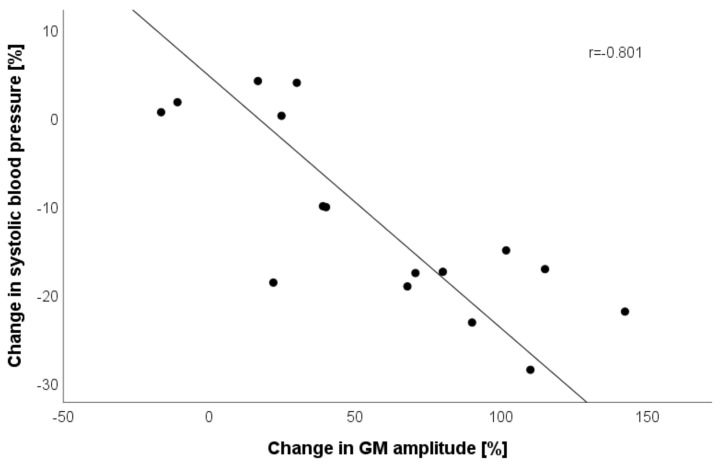
Pearson correlation (with linear regression line) between relative changes in gastrocnemius medialis (GM) amplitude and systolic blood pressure at post-rehabilitation when compared to baseline. Note: two cases missing due to poor data quality.

Lastly, we correlated changes in pooled GM and BF tensiomyographic parameters with changes in SBP and found a high correlation between GM Dm with SBP (r = −0.801; *p* < 0.001; Figure 3).

## 5. Discussion

The main finding of the present study with regard to cardiovascular and muscular parameters is that TM added to a standard rehabilitation program did not differentially affect recovery. Although systolic blood pressure significantly decreased and RR intervals increased between pre- and post-intervention measurements, and thus after four weeks of cardiac rehabilitation, no differences were found between the TM and control group.

Bokhari et al. [35] published a study in 2019 in which they reported significant improvements in the myocardial flow reserve (MFR) after 12 weeks, particularly in the groups that practiced TM (cardiac rehabilitation + TM (+20.7%), TM (+12.8%), cardiac rehabilitation (+5.8), control (−10.3%)).

Similar to the Bokhari et al. study, the mean duration of the studies mentioned in the introduction was 5.6 months (considering all cited studies that are related to cardiovascular parameters and that are not older than 1987 [9,13,16,17,18,19,21,22,23]). These results indicate that possible positive effects of practicing TM may unfold in the longer term.

Further, it should be mentioned that the myocardial flow reserve (MFR) was assessed with a 13N-ammonia positron emission tomography (PET). The assessment of MFR through a PET is considered quite a sensitive and specific (both are at about 90%) method to evaluate cardiovascular changes in patients suffering of chronic coronary syndrome [36,37,38]. This might explain why the authors found a significant effect despite the rather short intervention duration of 12 weeks.

The analysis of the tensiomyographic parameters showed a shift in two (out of three) skeletal muscles’ contractile properties towards a slow twitch phenotype (e.g., elongation of Tc), as was previously demonstrated by Šimunič et al. (2011) [39]. Additionally, consistent with previous findings, decreased muscle passive stiffness (e.g., increase in Dm) was also demonstrated [40,41]. However, no interaction between groups could be observed. In the pooled data we confirmed that baseline Tc and Dm of GM and BF negatively correlated to SBP, whereas increased GM Dm correlated to decreases in SBP after the rehabilitation period.

This was the first study that reported improvement in skeletal muscle contractile properties of three muscles after rehabilitation in cardiac patients. It was shown that improvements differed between muscles, with the greatest improvements in non-postural BF and postural distal GM, but not in postural proximal VL. The reasons for these findings remain unclear and thus warrant further investigations. Since no group effects or interactions were observed, we pooled the data of both groups and confirmed the baseline correlation with SBP, as shown in previous studies [31,32]. Blood pressure is the product of cardiac output and total peripheral resistance. A mediating factor for the association between a high number of type I fibers and low BP is that the greater the number of type I fibers, the lower the total peripheral resistance [42] where type I fibers, when compared to type II fibers have a higher number of capillaries surrounding the fibers [43]. Indeed, patients after heart failure have reduced microvascular density without decreased maximal aerobic capacity and increased fast type IIx muscle type [44]. After cardiac rehabilitation, increases in muscle function, exercise tolerance, and muscle aerobic capacity are regularly reported [45,46]. This is in line with our findings as it suggests that longer Tc, interpreted as higher amounts of type I fibers [39], correlates to blood pressure (specifically to SBP). Furthermore, we provided evidence that lower GM stiffness after rehabilitation is correlated to higher decreases in SBP. Muscle stiffness is another important contributor to higher SBP [47]. However, this was only evident in GM muscle, underlining the importance of muscle tone regulation in GM muscle for the regulation of blood pressure.

To quantify the effect of TM precisely, more intervention groups such as a control group, a rehabilitation group, a TM group, and a TM + rehabilitation group would be advisable. However, a plain control group that does not receive any rehabilitative care after suffering a cardiac event would not be ethically justifiable.

Finally, this pilot study is the first of its kind in Europe investing this topic and thus far is the second study worldwide. All testing has been precisely performed using state-of-the-art devices. Hence, the results and study structure can be used as a basis for further investigations.

### 5.1. Limitations

It is important to mention some factors that could be optimized. First, the rehab patients receive a multitude of interventions every day already. Beyond physical exercises, participants can also choose from a variety of relaxation methods. Second, adding further interventions to the rehab schedule, might in turn ironically induce stress, as patients are increasingly challenged to stick to their timetable. Third, when patients start with the rehab program they are commonly in quite poor condition. It seems likely that they will benefit exceedingly from any kind of rehabilitation intervention in succession to surgery or a cardiac event.

As previously discussed in detail above, the time factor appears to be the biggest limitation of our study. As a literature search revealed an average study duration of 5.6 months in the cited trials, this indicates that longer-term investigation of the effects of TM on the cardiovascular systems makes more sense.

We are aware of the fact that an adaption to the medication, the consumption of caffeinated drinks, and the consumption of nicotine could have considerable effects on the cardiovascular system and thereby the measured parameters. However, these disruptive factors can hardly be controlled, thus, we were unable to consider them in this trial. The easiest way to minimize this source of error is randomization (as we did) and a larger study population. Additionally, participants can be instructed to abstain from nicotine and caffeine (at least on the day of testing).

### 5.2. Conclusions

The results of our study indicate that TM, in addition to a standard cardiac rehabilitation program, does not differentially improve cardiac recovery after four weeks. However, as the literature suggests, positive effects may unfold in the longer term. Further investigations are needed to clarify if TM could affect cardiac rehabilitation outcomes positively in the long term.

## Figures and Tables

**Table 1:** Characteristics of the study groups and cause of cardiac rehabilitation: myocardial infarction (MI), acute coronary syndrome (ACS), percutaneous coronary intervention (PCI), and coronary artery bypass graft (CABG).

Characteristics	Control Group	TM Group	Total Sample
Male (*n*)	8 (89%)	8 (89%)	16 (89%)
Female (*n*)	1 (11%)	1 (11%)	2 (11%)
Age (years)	58.56 (±7.86)	59.78 (±7.84)	59.17 (±7.45)
Height (cm)	177.22 (±7.26)	173.44 (±7.73)	175.33 (±7.53)
Weight (kg)	95.44 (±10.41)	79.56 (±19.22)	87.50(±17.08)
BMI (kg/m^2^)	30.52 (±4.12)	26.31 (±5.10)	28.42 (±4.99)
Values are mean (±SD)			
**Cause of Cardiac Rehabilitation**			
MI	4 (44%)	3 (33%)	7 (39%)
ACS	1 (12%)	1 (12%)	2 (11%)
PCI	2 (22%)	2 (22%)	4 (22%)
CABG	2 (22%)	3 (33%)	5 (28%)

**Table 2:** Time course of the cardiac parameters in the control group and TM group of pre- and post-measurements.

Results Group Control & Group TM: Pre-Post													
Epochs												*p*-Value:	*p*-Value:
Parameter	Group	Baseline	Stand 1	Stand 2	Stand 3	Stand 4	Stand 5	Recovery 1	Recovery 2	Recovery 3	Recovery 4	Pre-Post	Pre-Post × Group
Heart rate [bpm]	Control pre (*n* = 9)	71 (±5)	84 (±8)	79 (±7)	80 (±7)	80 (±11)	81 (±12)	74 (±8)	73 (±9)	71 (±9)	70 (±8)	*p* = 0.058	*p* = 0.900
	Control post (*n* = 9)	72 (±10)	81 (±16)	78 (±13)	79 (±14)	77 (±13)	77 (±15)	72 (±8)	65 (±6)	65 (±6)	65 (±5)		
	TM pre (*n* = 9)	69 (±14)	75 (±14)	74 (±14)	78 (±15)	73 (±13)	79 (±18)	77 (±23)	67 (±12)	65 (±11)	65 (±12)		
	TM post (*n* = 9)	61 (±10)	74 (±8)	71 (±9)	71 (±9)	70 (±11)	70 (±10)	69 (±11)	61 (±10)	60 (±10)	60 (±11)		
RR-interval [ms]	Control pre (*n* = 9)	855 (±48)	727 (±55)	765 (±67)	768 (±67)	762 (±98)	758 (±108)	832 (±77)	843 (±96)	868 (±100)	874 (±79)	*p* = 0.050	*p* = 0.836
	Control post (*n* = 9)	891 (±91)	773 (±143)	791 (±129)	781 (±122)	804 (±122)	821 (±132)	863 (±102)	937 (±85)	938 (±75)	946 (±69)		
	TM pre (*n* = 9)	930 (±171)	823 (±147)	839 (±149)	805 (±140)	850 (±152)	805 (±139)	856 (±203)	929 (±165)	955 (±155)	958 (±176)		
	TM post (*n* = 9)	1009 (±146)	836 (±89)	870 (±116)	867 (±102)	887 (±122)	886 (±118)	904 (±156)	1010 (±179)	1032 (±177)	1023 (±171)		
Systolic blood pressure [mmHg]	Control pre (*n* = 9)	127 (±15)	129 (±17)	129 (±17)	129 (±20)	140 (±16)	139 (±18)	132 (±15)	131 (±12)	130 (±13)	125 (±16)	*p* = 0.012	*p* = 0.854
	Control post (*n* = 9)	116 (±13)	118 (±20)	118 (±19)	117 (±20)	129 (±13)	129 (±17)	126 (±26)	121 (±19)	118 (±15)	114 (±14)		
	TM pre (*n* = 9)	124 (±22)	123 (±21)	127 (±15)	126 (±19)	134 (±24)	136 (±23)	122 (±24)	122 (±21)	123 (±21)	126 (±22)		
	TM post (*n* = 9)	121 (±16)	106 (±20)	114 (±15)	116 (±14)	126 (±12)	126 (±17)	117 (±22)	120 (±18)	118 (±18)	124 (±19)		
Diastolic blood pressure [mmHg]	Control pre (*n* = 9)	81 (±12)	90 (±13)	91 (±13)	91 (±16)	94 (±8)	95 (±10)	75 (±6)	77 (±8)	76 (±7)	80 (±11)	*p* = 0.433	*p* = 0.928
	Control post (*n* = 9)	76 (±15)	89 (±15)	91 (±19)	93 (±19)	93 (±23)	91 (±30)	76 (±27)	72 (±20)	73 (±19)	75 (±16)		
	TM pre (*n* = 9)	82 (±15)	90 (±19)	93 (±15)	93 (±18)	93 (±21)	95 (±20)	70 (±11)	75 (±17)	76 (±18)	81 (±18)		
	TM post (*n* = 9)	80 (±8)	76 (±9)	83 (±4)	85 (±6)	89 (±7)	89 (±13)	73 (±14)	73 (±10)	74 (±11)	79 (±12)		
Mean arterial pressure [mmHg]	Control pre (*n* = 9)	100 (±13)	105 (±14)	105 (±14)	105 (±17)	112 (±11)	111 (±13)	96 (±8)	97 (±8)	97 (±9)	98 (±13)	*p* = 0.088	*p* = 0.754
	Control post (*n* = 9)	92 (±15)	95 (±23)	102 (±18)	104 (±17)	107 (±19)	105 (±25)	94 (±27)	91 (±20)	90 (±18)	91 (±14)		
	TM pre (*n* = 9)	99 (±19)	103 (±19)	106 (±15)	105 (±19)	109 (±22)	111 (±21)	95 (±23)	93 (±18)	94 (±19)	99 (±18)		
	TM post (*n* = 9)	97 (±11)	87 (±12)	94 (±7)	97 (±8)	104 (±8)	103 (±13)	89 (±16)	92 (±12)	91 (±13)	97 (±14)		
Stroke index [mL/m^2^]	Control pre (*n* = 9)	31 (±7)	28 (±4)	27 (±4)	27 (±2)	26 (±4)	26 (±4)	37 (±7)	38 (±7)	38 (±7)	32 (±8)	*p* = 0.797	*p* = 0.839
	Control post (*n* = 9)	32 (±7)	29 (±4)	27 (±4)	26 (±2)	27 (±4)	26 (±4)	35 (±7)	37 (±7)	39 (±7)	34 (±8)		
	TM pre (*n* = 9)	33 (±4)	30 (±5)	32 (±5)	31 (±4)	31 (±5)	31 (±6)	34 (±4)	38 (±6)	39 (±8)	32 (±6)		
	TM post (*n* = 9)	35 (±7)	32 (±4)	30 (±5)	30 (±5)	31 (±5)	30 (±5)	36 (±7)	38 (±8)	39 (±8)	35 (±8)		
Cardiac index [l/(±min·m^2^)]	Control pre (*n* = 9)	2.2 (±0.4)	2.3 (±0.4)	2.1 (±0.3)	2.2 (±0.3)	2.1 (±0.4)	2.1 (±0.3)	2.7 (±0.2)	2.8 (±0.6)	2.7 (±0.5)	2.2 (±0.4)	*p* = 0.217	*p* = 0.822
	Control post (*n* = 9)	2.3 (±0.9)	2.3 (±0.5)	2.1 (±0.3)	2.1 (±0.2)	2.0 (±0.3)	2.0 (±0.3)	2.6 (±0.6)	2.4 (±0.6)	2.6 (±0.8)	2.2 (±0.7)		
	TM pre (*n* = 9)	2.3 (±0.6)	2.3 (±0.8)	2.4 (±0.7)	2.5 (±0.7)	2.3 (±0.6)	2.4 (±0.6)	2.6 (±0.8)	2.5 (±0.6)	2.6 (±0.8)	2.1 (±0.6)		
	TM post (*n* = 9)	2.1 (±0.5)	2.4 (±0.5)	2.1 (±0.4)	2.1 (±0.3)	2.1 (±0.4)	2.1 (±0.4)	2.5 (±0.6)	2.3 (±0.6)	2.3 (±0.5)	2.1 (±0.5)		
TPRI [dyne·s·m^2^/cm^5^]	Control pre (*n* = 9)	3670 (±597)	3595 (±813)	3832 (±712)	3838 (±930)	4269 (±851)	4311 (±710)	2920 (±617)	2855 (±609)	2977 (±646)	3682 (±799)	*p* = 0.308	*p* = 0.769
	Control post (*n* = 9)	3602 (±1183)	3323 (±767)	3707 (±808)	3938 (±591)	4209 (±821)	4328 (±903)	3042 (±914)	3074 (±910)	2952 (±969)	3465 (±1029)		
	TM pre (*n* = 9)	3833 (±1350)	3751 (±1585)	3806 (±1424)	3568 (±1330)	4028 (±1622)	4033 (±1524)	2955 (±826)	2971 (±955)	3037 (±1053)	3982 (±1500)		
	TM post (*n* = 9)	3850 (±1328)	3037 (±850)	3634 (±602)	3788 (±605)	4129 (±851)	3952 (±901)	3019 (±995)	3238 (±918)	3223 (±826)	3832 (±1328)		

Note: analyses based on log-transformed data.

**Table 3:** Change in tensiomyographic parameters of control group and transcendental meditation (TM) group at pre and post measurements.

Parameter	Group	Pre	Post	p_TIME_ (η^2^)	p_TIME × GROUP_
Vastus lateralis					
Contraction time (ms)	Control (*n* = 9)	25.7 (±6.3)	27.4 (±1.8)	0.133	0.787
Contraction time (ms)	TM (*n* = 9)	25.7 (±3.2)	28.1 (±3.8)		
Amplitude (mm)	Control (*n* = 9)	4.80 (±1.87)	4.97 (±1.41)	0.363	0.855
Amplitude (mm)	TM (*n* = 9)	6.11 (±1.41)	6.36 (±1.57)		
Gastrocnemius medialis					
Contraction time (ms)	Control (*n* = 9)	21.6 (±3.6)	26.6 (±4.9)	<0.001 (0.712)	0.634
Contraction time (ms)	TM (*n* = 9)	24.7 (±4.9)	28.9 (±6.8)		
Amplitude (mm)	Control (*n* = 9)			0.008 (0.524)	0.305
Amplitude (mm)	TM (*n* = 9)				
Biceps femoris					
Contraction time (ms)	Control (*n* = 9)	35.6 (±8.2)	44.1 (±5.4)	<0.001 (0.764)	0.405
Contraction time (ms)	TM (*n* = 9)	40.9 (±10.9)	47.1 (±9.3)		
Amplitude (mm)	Control (*n* = 9)	5.91 (±3.31)	7.99 (±2.16)	<0.001 (0.714)	0.151
Amplitude (mm)	TM (*n* = 9)	7.85 (±5.45)	11.80 (±5.78)		

**Table 4:** Correlation between tensiomyographic parameters and systolic blood pressure.

Parameter	Contraction Time	Amplitude
Vastus lateralis	r = 0.139; *p* = 0.683	r = −0.088; *p* = 0.798
Gastrocnemius medialis	r = −0.631; *p* = 0.037	r = −0.707; *p* = 0.015
Biceps femoris	r = −0.522; *p* = 0.099	r = −0.703; *p* = 0.016

## Data Availability

Data is contained within article.
